# Ureteral Orifice Detection in Ureteroscopic Images Based on Large-Kernel Convolutional Neural Networks and Attention-Based Feature Fusion

**DOI:** 10.3390/bioengineering13040459

**Published:** 2026-04-14

**Authors:** Liang Li, Chen-Yi Jiang, Xing-Jie Wang, Yuan-Jun Wang, Jian Zhuo

**Affiliations:** 1 School of Health Science and Engineering, University of Shanghai for Science and Technology, Shanghai 200093, China; 2 Department of Urology, Shanghai General Hospital, Shanghai Jiao Tong University School of Medicine, Shanghai 200080, China

**Keywords:** large-kernel CNNs, ureteral orifice detection, object detection

## Abstract

**Objective**: To enhance the information modeling capacity of large-kernel convolutional neural networks and to build a ureteral orifice detection framework for ureteroscopic imaging. **Methods**: A retrospective dataset of ureteroscopic images from 222 patients was collected. The patients were randomly divided into training and testing sets at a ratio of 7:3. Initially, video files were converted into image frames, and feature-relevant images were manually labeled by physicians. Subsequently, a ConvNeXt-based backbone augmented with squeeze-and-excitation (SE) modules was employed to extract diverse deep features. SCConv modules were incorporated across stages to strengthen the network’s feature extraction performance. Lastly, enhanced spatial excitation attention mechanisms were cascaded to achieve superior feature fusion and detection accuracy. Comparative experiments were conducted against baseline models, including ConvNeXt, assessing accuracy, computational overhead, and inference latency. **Results**: On a test set of 491 ureteroscopic images, all models achieved mAP@50 values above 0.75, whereas the proposed network achieved 0.890, markedly exceeding baseline performance. The model operated at 20 ms per frame, achieving a frame rate of 50 FPS. **Conclusions**: We developed an improved deep learning framework based on large-kernel convolutional networks for real-time ureteral orifice detection in endoscopic scenarios. This system achieves a favorable balance between detection accuracy and real-time efficiency. The method demonstrates significant potential as a training and feedback tool for residents and junior urologists in clinical environments.

## 1. Introduction

The ureteral orifice is of critical importance in urological procedures, and its localization and assessment typically require substantial clinical expertise and focused attention from surgeons [1,2]. Examination of the morphology of the ureteral orifice and subsequent biopsy can assist in determining whether lesions are due to calculi, tuberculosis, inflammation, stricture, or benign or malignant tumors, which is valuable for identifying the nature and location of upper urinary tract diseases. When performing rigid retrograde ureteroscopy for pigtail catheter insertion, precise and prompt identification of the ureteral orifice position and trajectory improves surgical efficiency [3,4], decreases operative duration, and mitigates the likelihood of stent displacement. Additionally, precise identification of the ureteral orifice under challenging surgical visualization can help avoid accidental damage and minimize complications, a scenario frequently encountered during cystoscopy and transurethral resection of the prostate. Consequently, precise evaluation of the ureteral orifice condition is essential for safe and effective urological surgical practice [5,6,7].

The ureteral orifice is anatomically fixed at the posterolateral angle of the trigone, yet its identification during surgery is often challenging due to multiple factors [8]. Physiological contractions periodically reduce the size of the orifice, thereby complicating its detection during rapid exploration. On the other hand, ureteroscopy typically relies on white-light illumination, and suboptimal viewing angles or a relatively flat bladder wall may introduce shadows from vessels and lighting, impairing overall judgment [4]. In ureteroscopic examinations, a surgeon’s experience extends beyond insertion depth control to include distinguishing the ureteral orifice from multiple interfering factors in complex operative settings. Identification often requires tentative mechanical probing with the endoscope, which itself may cause injury to the patient. The drawbacks of this approach are further amplified during procedures involving high-energy instruments. In these scenarios, exogenous contrast agents are often required for visual guidance, leading to increased preoperative time and concerns regarding reagent safety and cost [9,10]. Therefore, developing an image-based ureteral orifice detection algorithm could be beneficial in such scenarios [11], improving surgical efficiency and quality while supporting the training of novice urologists.

Although artificial intelligence has been increasingly applied in medicine in recent years, comprehensive research in urology—especially within intraoperative endoscopic scenarios—remains limited and warrants deeper exploration. Existing studies have primarily focused on imaging-assisted analysis and surgical decision support for urinary calculi, while automated recognition of key endoscopic anatomical structures is still at an early stage [12,13]. Lazo and colleagues introduced a deep learning approach for lumen segmentation in ureteroscopic images to support intraoperative navigation and path recognition [14]. Wang C et al. developed the AiFURS system, enabling real-time object detection and quantitative analysis during flexible ureteroscopic surgery, representing a significant step toward clinical validation of endoscopic AI [15]. Nevertheless, substantial gaps remain in the application of AI to urological endoscopy involving lumen-related analysis [16]. For ureteral orifice identification, Peng X and colleagues pioneered the application of deep learning to real-time detection in ureteroscopic videos, presenting an SSD-based automated approach for fast localization [17]. In subsequent work, Liu [18] integrated object detection with a tracking system to recognize key anatomical structures of the ureteral orifice in urinary endoscopic videos. To achieve real-time monitoring, a combination of low-frequency detection and target tracking was adopted, which enabled continuous region-of-interest labeling but introduced a high-risk trade-off in sustained target detection due to reliance on tracking algorithms. As this part of the work was conducted at an earlier stage, the shallow convolutional architecture of SSD was not well suited to handle low-contrast and large-scale variations under structurally ambiguous endoscopic images; therefore, higher-resolution and relatively stable image data were used for analysis. Furthermore, due to limited research progress at that time, techniques such as attention mechanisms and feature pyramids had not yet been widely incorporated into object detection tasks, making the comparative framework of this work appear conservative in light of current advancements. Lastly, because earlier data collection relied largely on multiple video recordings of the same cases across different platforms, the total number of distinct cases was limited, leading to concerns regarding the statistical robustness and generalizability of the findings, even in a medical setting. In response to these limitations, we propose further exploration aimed at achieving robust real-time monitoring, thereby improving the stability and accuracy of detection.

Reviewing the evolution of object detection networks, as clinical application scenarios continue to expand, algorithmic performance faces similar challenges, including small-object detection [19], ambiguous foreground–background boundaries [20], stringent requirements for real-time performance and stability, and limited yet complexly distributed datasets [21]. During the transition from handcrafted features to deep learning, most methods adopted sliding-window concepts and were unified under convolutional neural network frameworks [22]. Subsequently, methods evolved from R-CNN to the canonical two-stage detector framework, which combines feature extraction with classification and regression [23]. However, amid intense competition, SSD introduced anchor-based single-stage detection [24], and YOLO [25] further adopted and integrated this concept to become a benchmark for next-generation image detection. With continued development, numerous techniques were incorporated into these frameworks, including, but not limited to Focal Loss [26], anchor-free methods [27], and NMS [28], all of which further enhanced the performance of single-stage detectors. Later, the adoption of Transformers in computer vision enabled models like DETR [29] to excel on large datasets, yet they have not exhibited substantial performance gains in downstream tasks characterized by scarce and challenging clinical data. At the same time, RepLKNet [30] revived interest in traditional CNN paradigms by advocating the use of large convolutional kernels, shifting focus away from Transformer-based models. With models such as ConvNeXt V2 [31] further refining the large-kernel concept across training strategies and architectural design, their strong complementarity with attention mechanisms and favorable engineering and deployment characteristics have renewed interest in traditional CNNs.

In this work, a dataset was established from clinical ureteroscopic images, and effective ureteral orifice detection was achieved through the improvement and training of a traditional large-kernel convolutional network. The key contributions of this work are summarized as follows:

(1) By combining large-kernel convolutions with spatial–channel attention mechanisms, a feature extraction backbone suitable for clinical medical image data was designed.
(2) Based on the performance characteristics of the feature extraction network, the feature fusion stage was improved by incorporating channel attention mechanisms, resulting in a significant enhancement of overall performance.
(3) Extensive clinical data were collected to evaluate the proposed improvements, successfully validating the robustness and accuracy of the network architecture.

## 2. Materials and Methods

**Figure 1:**
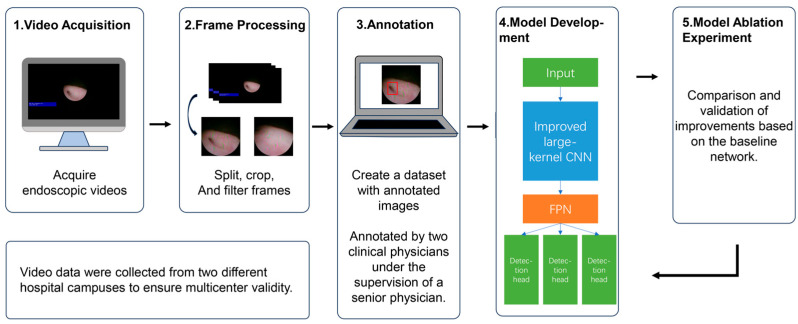
Research Flowchart.

This study constructed a UO object detection model based on ureteroscopic images, involving four main steps: data collection, image extraction, feature annotation, and model training. The research workflow is shown in Figure 1.

First, endoscopic videos are acquired, and those with clear features are selected; second, the videos are split into individual frames, cropped, and further filtered. Third, software is used to annotate the images and create a dataset. Finally, various models are developed to determine whether the ureteral opening is present in the images and to identify its location. Model performance is evaluated using quality metrics such as specificity and sensitivity, as well as time metrics, including computational cost and detection time.

### 2.1. Patients

Biopsy case data were retrospectively collected from patients at Shanghai General Hospital from October 2022 to June 2024. To ensure the scientific validity of the study, patients were sourced from two campuses of the hospital and their data were mixed to create the dataset. Inclusion criteria: Patients whose ureteral orifices have intact physiological contours, without infectious diseases or comorbid infectious conditions. Exclusion criteria: (1) Patients who had undergone procedures such as stent implantation, providing exogenous markers. (2) Patients whose physiological contour was compromised due to surgical damage or other causes.

### 2.2. Data Extraction

All video recordings were screened by a senior clinician with more than ten years of experience to assess compliance with exclusion criteria prior to dataset preparation. First, the pixel start and end positions of the endoscopic field of view within each video were identified to define the cropping origin and spatial extent. Next, the MP4 video files were imported into MATLAB R2022a, and frame information was extracted for subsequent image processing. Finally, each frame was iteratively cropped according to the predefined spatial parameters and exported as PNG images.

### 2.3. Feature Annotation

Under the guidance of clinical physicians, all images were annotated using the LabelMe software (version 3.16.7) in YOLO format, with labels stored as TXT files. The regions of interest were required to include the complete ureteral orifice lumen and its surrounding anatomical contours without any occlusion. In total, 1939 images were extracted from ureteroscopic videos of 222 cases. Data partitioning was performed based on different hospital campuses. Specifically, the videos from the Songjiang District campus of Shanghai General Hospital, which were more abundant, were selected as the training set, while the dataset from the Hongkou District campus, following data cleaning and classification, was designated as the validation set, yielding an overall approximate ratio of 7:3. The training set consisted of 1448 images, while the validation set contained 491 images. Model performance was evaluated based on the alignment between the predicted region center and the region of interest, as well as the overlap between predicted and annotated regions. Please refer to Table 1 for details of the dataset.

**Table 1:** Dataset partitioning.

Part	Color Format	Picture Format	Case	Figure
Train	RGB	PNG	154	1448
Val	RGB	PNG	68	491

### 2.4. Model Establishment and Evaluation

**Figure 2:**
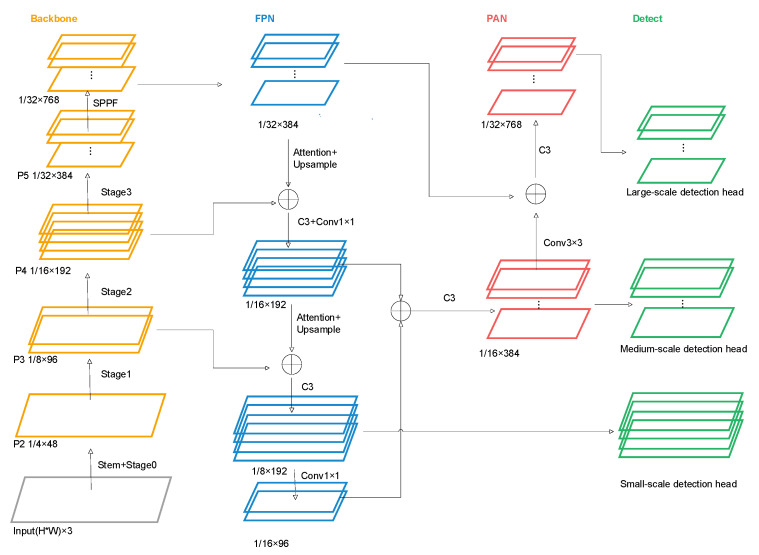
Schematic diagram of the network architecture.

In ureteroscopic images, ureteral orifice targets are typically characterized by small pixel size, significant morphological variation, and low boundary contrast, while the surrounding regions often contain abundant structurally similar background textures and specular reflections. Networks optimized for SOTA tasks often depend on local receptive-field-based feature extraction, which can result in target–background ambiguity; establishing relational modeling between the target and its surrounding context helps compensate for local texture noise and improves robustness and generalization. During experiments using ConvNeXtV2 with large convolutional kernels as the baseline network, we observed excessive redundant information and insufficient focus on salient features for small-sample targets, which posed significant challenges to final performance. Accordingly, we improved the baseline blocks by increasing channel depth and applying global channel recalibration, facilitated cross-stage communication using fused global spatial and local contextual information, and employed channel attention guided by inter-channel structural priors within the Feature Pyramid Network, thereby achieving robust small-target detection under complex medical conditions with limited samples. The schematic diagram of the proposed network architecture is shown in Figure 2.

#### 2.4.1. Block

**Figure 3:**
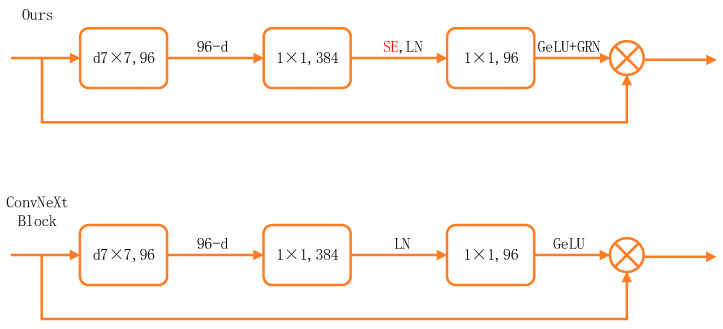
Schematic comparison of the proposed block and the original block.

The proposed network builds upon ConvNeXt and forms stages through repeated basic blocks, implying that modifications to individual blocks can significantly influence the performance of the entire architecture. In the baseline design, complexity is reduced at the expense of accuracy to align with SOTA benchmarks and ensure broader applicability and comparability; however, this tendency should be specifically addressed in downstream tasks. To achieve better performance in clinical imaging scenarios and obtain reliable results, adding and replacing architectural components have proven highly effective across extensive experiments. This study optimizes the ConvNeXt architecture by drawing inspiration from RepLKNet. To mitigate redundancy introduced by the enlarged receptive field of large convolution kernels and improve task performance, a Global Response Normalization (GRN) module is incorporated to enhance channel feature diversity. Meanwhile, to counteract the accuracy degradation caused by depthwise separable convolutions, SE attention is employed to model inter-channel relationships and compensate for this limitation. The structure of the basic module is illustrated in Figure 3.

**Figure 4:**
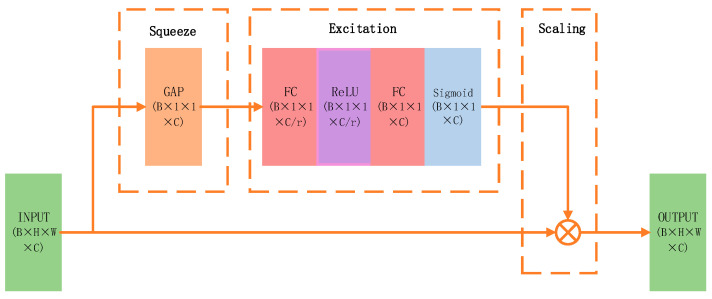
Illustration of the Squeeze-and-Excitation (SE) structure.

The incorporation of the Squeeze-and-Excitation (SE) attention mechanism aims to overcome the uniform importance assigned to different channel features by large convolutional kernels, increasing sensitivity to semantically relevant cues for target recognition through inter-channel dependency modeling and dynamic feature reweighting, ultimately enhancing performance. A detailed schematic is provided in Figure 4.

The underlying principle is described as follows, assuming that the output feature map of the large-kernel convolution block is given by:

(1) $$X\in R^{C\times H\times W},$$

After the squeeze operation, the following representation is obtained:

(2) $$z_{c}=\left(\frac{1}{\left(H\times W\right)}\right)\cdot \sum_{i=1}^{H}\sum_{j=1}^{W}X_{c\left(i,j\right)},$$

Since $X_{c}(i,j)$ represents the feature map of a specific channel, the vector $z\in R^{C}$ encodes the global response intensity of each channel. After applying the excitation operation, the following formulation is obtained:

(3) $$s=\sigma \left(W_{2}\cdot \delta \left(W_{1}\cdot z\right)\right),$$

Here, δ denotes the ReLU activation function, σ represents the Sigmoid function, and s corresponds to the channel-wise attention coefficients. Finally, the channel-wise weights are applied back to the original feature map:

(4) $${\tilde{X}}_{c}=s_{c}\cdot X_{c},$$

This enhancement suppresses redundant responses without significantly increasing computational complexity, thereby facilitating the identification of critical targets.

#### 2.4.2. Stage

Although the reconstruction of the basic blocks effectively reduces feature redundancy, duplicated redundant information remains across different stages. Following the principle of differentiated utilization of redundant and salient information, we further introduce Spatial and Channel Reconstruction Convolution (SCConv) at the end of each stage, and the corresponding mechanism is described below.

**Figure 5:**
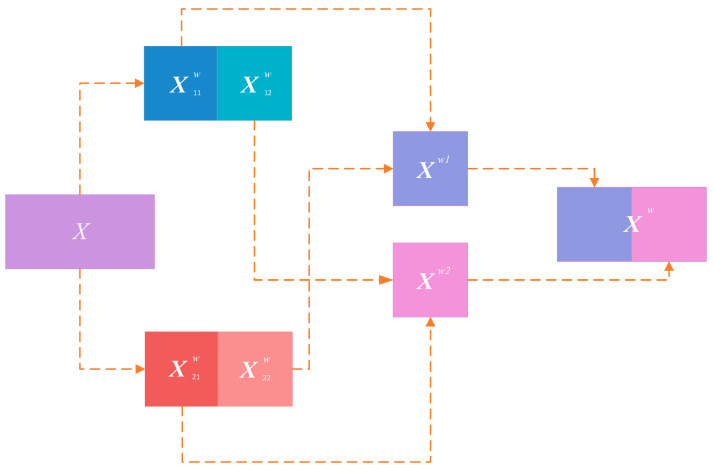
Schematic diagram of the Spatial Reconstruction Unit.

First, the original feature map is reconstructed by suppressing spatial redundancy; specifically, a binary mask W is computed to divide the features into information-rich components $W_{1}$ and information-redundant components $W_{2}$, which are then multiplied element-wise with the original feature map X to obtain two processed feature maps. These are subsequently split into two parts, summed element-wise, and concatenated to form a new feature map, as illustrated in Figure 5.

Following the generation of $X^{w}$, channels are split into an upper branch $X_{up}$ and a lower branch $X_{low}$ to further compress redundant channel information. The upper branch adopts a computationally intensive yet high-capacity feature extraction strategy:

(5) $$Y_{1}=M^{G\left(X_{up}\right)}+M^{P1\left(X_{up}\right)},$$

In contrast, the lower branch performs feature reuse with relatively low computational cost:

(6) $$Y_{2}=M^{P2\left(X_{low}\right)}\cup X_{low},$$

Subsequently, global average pooling is applied to both outputs to obtain channel-wise statistical vectors:

(7) $$S_{m}=\left(\frac{1}{\left(H\times W\right)}\right)\sum_{i=1}^{H}\sum_{j=1}^{W}Y_{c\left(i,j\right)},\;m\in \left\{1,2\right\},$$

Subsequently, the weighting coefficients of the two branches are calculated as:

(8) $$\beta_{1}=\frac{e^{s_{1}}}{\left(e^{s_{1}}+e^{s_{2}}\right)},\;\beta_{2}=\frac{e^{s_{2}}}{\left(e^{s_{1}}+e^{s_{2}}\right)},$$

Finally, the weighted sum yields the output:

(9) $$Y=\beta_{1}\cdot Y_{1}+\beta_{2}\cdot Y_{2},$$

#### 2.4.3. Feature Pyramid Network (FPN)

**Figure 6:**
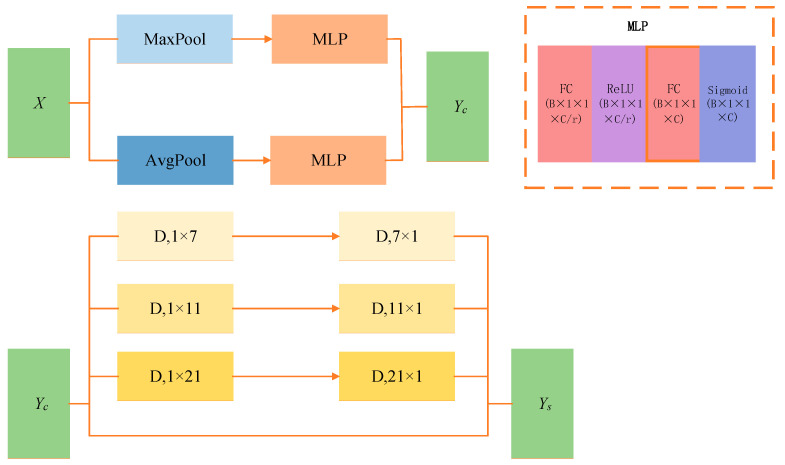
Schematic diagram of the Prior-Guided Depth-wise Attention mechanism.

After improving the backbone network, we attempted to apply the same architectural modifications consistently to components within the feature pyramid; however, repeated experiments yielded unsatisfactory results. To address this issue, we enhanced the standard spatial–channel attention mechanism by incorporating prior constraints, as schematically shown in Figure 6.

First, the original feature map X is processed by average pooling and max pooling, and the resulting vectors are transformed using a Multi-Layer Perceptron (MLP), as in the Convolutional Block Attention Module (CBAM) [32]:

(10) $$Y_{c}=\sigma \left(MLP\left(AvgPool\left(X\right)\right)+MLP\left(MaxPool\left(X\right)\right)\right),$$

Here, σ denotes the Sigmoid function, and the MLP follows the formulation in Equation (3), after which the feature map is passed through depth-wise convolutions with multiple branches at different scales to capture multi-scale spatial relationships and is finally fused by element-wise summation:

(11) $$Y_{s}=Conv_{\left(1\times 1\right)}\left(\sum_{i=0}^{3}Branch_{i}\left(DwConv\left(Y_{c}\right)\right)\right),$$

In this context, DwConv denotes depth-wise convolution, and each branch corresponds to a multi-scale spatial modeling path, where i=0 indicates an identity connection.

## 3. Experimental Settings and Evaluation

All experiments in this study were conducted on the same hardware and software platform to ensure a consistent experimental environment. The system was equipped with an NVIDIA RTX 4090 GPU, an Intel Core i9-13900KF CPU, and 32 GB of DDR5 RAM. All images were subjected to default data augmentation before training and resized to 640 × 640 PNG format; since no pretraining was applied, the number of training epochs was set to 300 and the batch size to 8.

To comprehensively evaluate model performance in ureteral orifice detection, this study adopts commonly used metrics in object detection, including precision (Pre), recall (Rec), and mean average precision (mAP). The aforementioned metrics are derived from four basic elements: true positive (TP), false positive (FP), false negative (FN), and true negative (TN).

Here, TP denotes that the predicted bounding box successfully matches the ground truth bounding box of the ureteral orifice; FP indicates that a bounding box is predicted but does not match any true target; TN denotes that no detection box is produced by the model in images without targets; and FN indicates that a true target exists but is missed by the model. Based on the above statistics, precision and recall can be defined as follows:

(12) $$Precision=\frac{TP}{TP+FP},$$

(13) $$Recall=\frac{TP}{TP+FN},$$

The mean Average Precision (mAP) is a widely used metric in object detection that computes the average of the Average Precision (AP) values across all classes, thereby providing a single scalar measure of overall model performance, as defined by the following expression:

(14) $$mAP=\frac{\sum_{q=1}^{Q}AveP\left(q\right)}{Q},$$

where *Q* is the number of queries in the set, and *q* is the average precision of a query. mAP@0.5 represents the proportion of accuracy with bounding boxes overlapping with the ground truth by 50%.

## 4. Results and Discussion

### 4.1. Ablation Experiments

First, we conducted ablation experiments to evaluate the overall network improvements, with all experiments performed on a ureteroscopic image dataset, in which we individually incorporated SE, SCConv, and a prior-constrained attention mechanism, as well as the combined use of SE and SCConv, and finally compared these configurations with the fully improved model, details of the ablation study are provided in Table 2, demonstrating that single modifications or repetitive stacking of an individual improvement yield markedly inferior performance compared with the combination of all three enhancements, thereby preliminarily validating that attention strategies at different network locations and the prioritization of critical information cannot be addressed through simple repetition or uniform treatment.

**Table 2:** Ablation experiments.

Module	*p*-Value	R-Value	mAP@0.5
Baseline	0.792	0.804	0.839
Baseline + SE	0.848	0.800	0.856
Baseline + SCConv	0.831	0.749	0.831
Baseline + FPN Attention	0.851	0.777	0.878
Baseline + SE + SCConv	0.823	0.815	0.864
Baseline + SE + FPN Attention	0.850	0.809	0.876
Baseline + SCConv + FPN Attention	0.817	0.817	0.853
Ours	0.838	0.825	0.890

**Figure 7:**
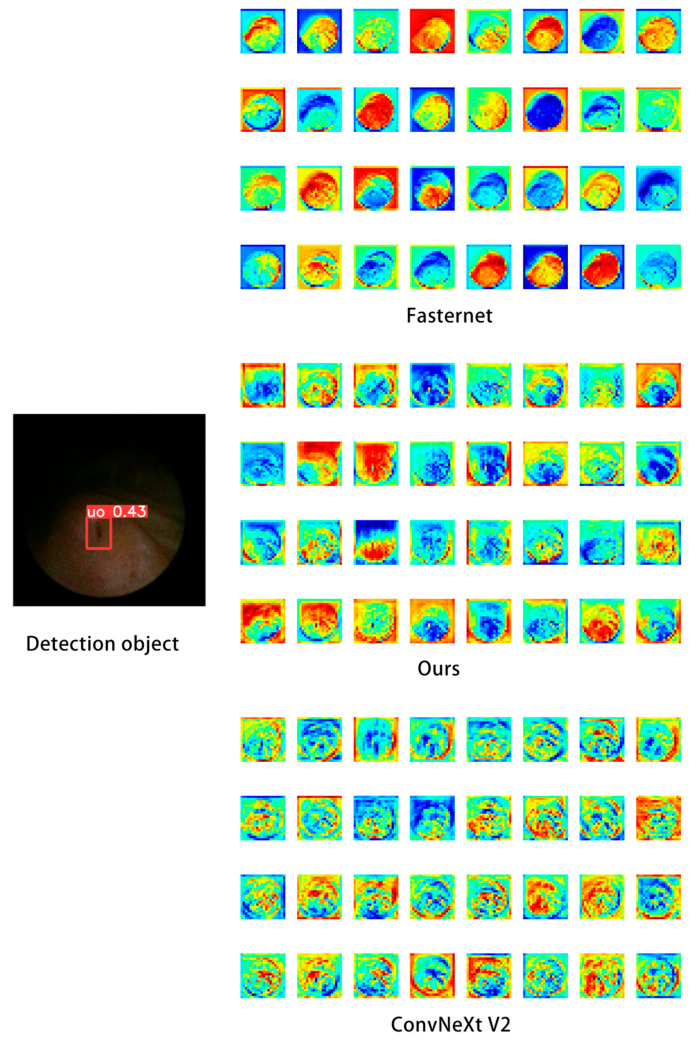
Feature extraction comparison map.

Compared with widely used YOLO models or the large-kernel baseline ConvNeXtV2, our backbone network exhibits superior capability in global feature extraction, enabling it to capture more informative features from the same image while maintaining rich channel responses without significant spatial drift, thereby consistently localizing sharp boundary variations around the target regions. Since the backbone is designed to iteratively emphasize critical features while suppressing redundant information, we performed an experimental comparison of backbone-level feature extraction on selected images, as shown in Figure 7.

### 4.2. Comparison Experiments

Meanwhile, in the feature fusion stage, we introduced several modifications to conventional spatial–channel excitation attention mechanisms; accordingly, extensive comparative experiments were conducted for this component. Specifically, we replaced different recently introduced attention mechanisms at identical positions within the network and conducted a comparative analysis of their performance. Considering the overall effectiveness, Prior-Guided Depthwise Attention exhibits the most well-balanced performance, as reported in Table 3.

During the process of data accumulation, we constructed multiple datasets, as summarized in Table 4.

**Figure 8:**
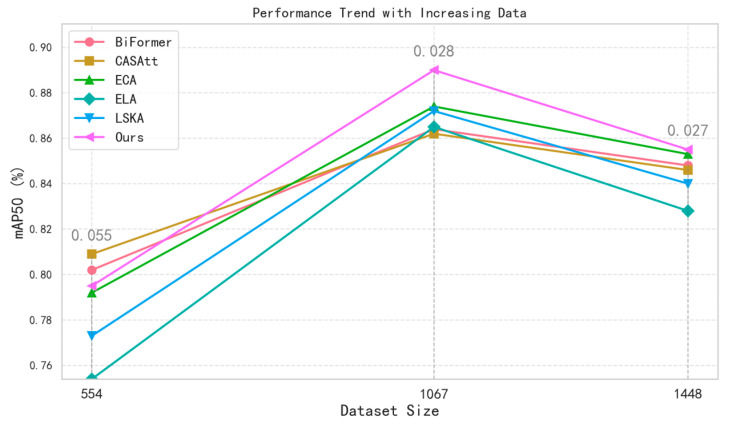
Comparison experiment results under different sample sizes.

The aforementioned attention mechanisms were evaluated across the different datasets, and the results were further processed and summarized in Figure 8. In the figure, different legends correspond to different attention mechanisms, and the difference between the maximum and minimum mAP@50 values within each dataset is annotated above each group, indicating that, as the dataset size increases, the overall performance trend becomes more stable, while our model consistently achieves superior results.

**Figure 9:**
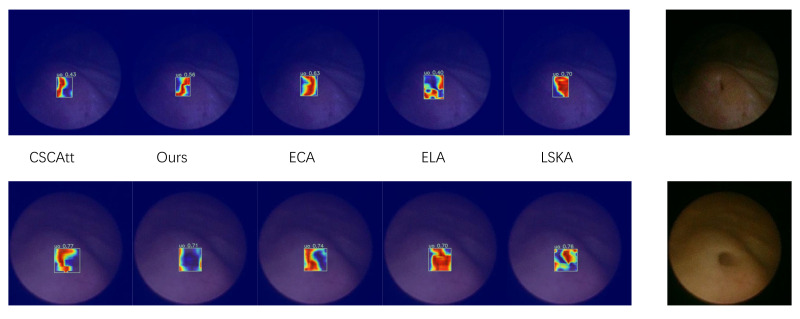
Detection heatmap visualization. The ureteral orifice under a fully dilated condition (**lower panel**) and a non-fully dilated condition (**upper panel**).

Furthermore, we experimentally validated the practical impact of the feature fusion module on object detection; as shown in Figure 9, we performed a localized comparison of the feature maps extracted by the detection head using heatmap visualizations. It can be observed that the adopted attention mechanism delineates the target boundaries more accurately on the feature maps without encroaching upon the target interior, and the global heatmap gradients demonstrate comparatively smooth and well-behaved characteristics.

**Figure 10:**
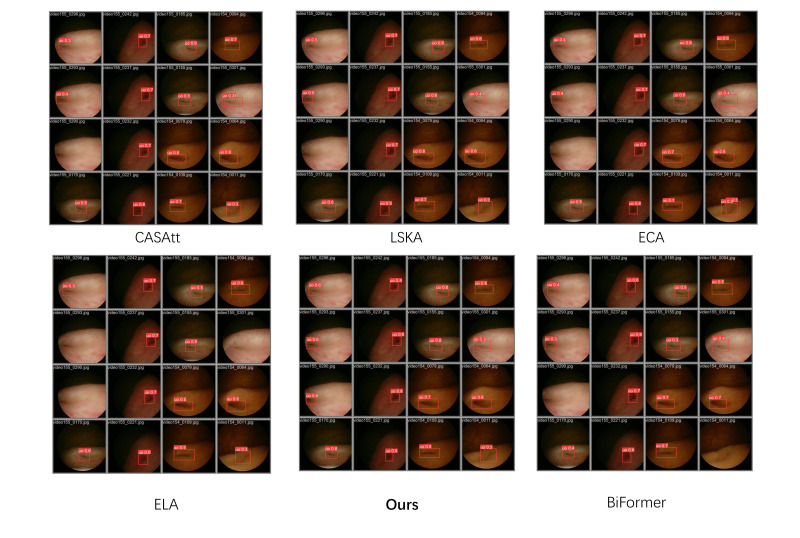
Comparative visualization of detection results of different models on the validation set.

Finally, we compared the performance of different network architectures on the validation set, as shown in Figure 10, demonstrating that our proposed structure achieves favorable results in terms of both detection accuracy and corresponding recall. When trained on the same data, the proposed method demonstrates superior robustness and generalizability in challenging environments, suggesting enhanced practical effectiveness in clinical ureteral orifice detection applications.

We also performed a horizontal comparison of the current object detection networks. Because we employed the open-source versions of each network, we conducted three trials with random initialization to compute the mean and variance of mAP@50, thereby evaluating the networks’ performance. As shown in Table 5, our model outperforms mainstream SOTA object detection models at the m-level, achieving better overall results with lower computational cost, as evidenced by both the mean mAP@50 and its variance.

**Figure 11:**
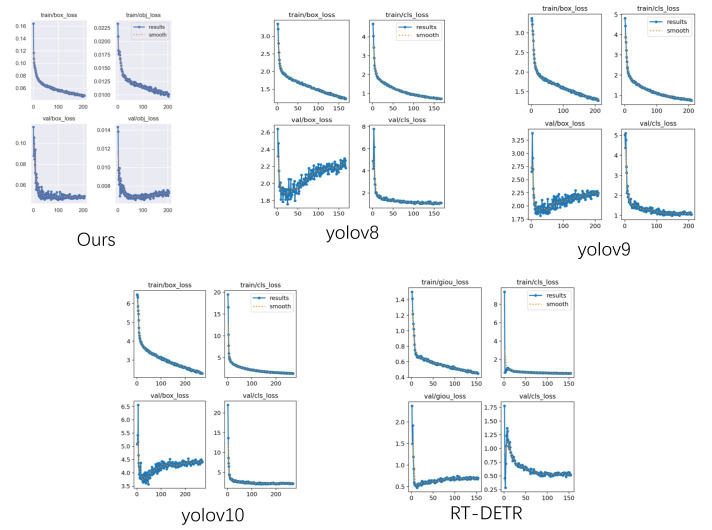
Model Loss Function Variation Plot.

Despite this, we note that our results on the finer-grained mAP@0.5–0.95 metric are unsatisfactory, and the metric scores poorly relative to all models in the cross-model comparison. Since this metric shows weak correlation with the other three accuracy measures, we investigated the convergence characteristics of different loss functions. Figure 11 shows that all model loss functions follow a consistent trend, indicating that the dataset itself is sound. Regarding Box-Loss, the loss associated with bounding-box size exhibits pronounced oscillations. As illustrated in Figure 10, the sizes of predicted bounding boxes fluctuate markedly between algorithms, indicating the inherent ambiguity in defining a fully complete detection boundary. The ureteral orifice presents a boundary distinct from that of polyps or tumors due to its directional morphology; although we imposed a selection criterion during dataset creation, it serves primarily to aid clinicians in annotation rather than to reflect an absolute biological standard. Consequently, clinicians tend to enlarge bounding boxes to make annotations more comprehensive, which is a key factor limiting the achievable high-precision range.

## 5. Discussion

As described previously, clinical images in object detection tasks are often associated with various challenges, and in exploring this domain using a large-receptive-field-enhanced CNN, this study introduced attention mechanisms and integrated established object detection techniques to redesign the network architecture, achieving favorable results.

First, we performed cross-validation analyses of individual components through systematic ablation studies. The experimental results show that incorporating a single module is insufficient to achieve the final optimized performance, and the isolated introduction of SCConv even caused degradation relative to the baseline. This may be attributed to the insertion of a single attention mechanism between stages, which may cause critical structural information to be overlooked. Although paired combinations of multiple attention modules still fall short of the final optimized scheme, an overall improvement in recall can be observed compared with introducing a single module, suggesting that information from ambiguous structures may be extracted under the joint influence of multiple attention mechanisms.

Feature map comparisons further support this inference that the proposed design facilitates the extraction of ambiguous structural information. From the original images, the circular region corresponds to the area where informative content begins to appear, whereas the area outside the circle is a purely black cropped margin containing no useful information; therefore, the desired behavior is to concentrate gradient variations within the circular region rather than along its boundary, since the true detection target lies in the information-rich central region rather than the sharp intensity transition at the image border. Accordingly, our method captures more useful information in the feature maps and avoids emphasizing gradients along these misleading borders. This improvement can be attributed to the attention mechanism, which effectively suppresses redundant responses.

After confirming that the overall optimization scheme exhibits sufficient robustness, we devoted substantial effort to the feature fusion stage to verify two hypotheses: (1) the proposed prior-guided attention strategy can effectively improve overall performance, and (2) the improved backbone can achieve faster and more stable convergence under small-sample conditions, addressing the limitation of insufficient clinical images. In comparative experiments, our network achieved the best mAP@50 among all evaluated attention mechanisms, while also maintaining a favorable balance between recall and precision. Furthermore, experiments across datasets of different sizes validated our second hypothesis: under extremely limited data, large performance gaps existed among attention mechanisms, whereas when the number of cases increased to 155 and 222, the difference between the maximum and minimum mAP@50 values became nearly unchanged, indicating that our network improvements enable stable performance even with fewer samples.

Finally, evaluations on real clinical images demonstrated promising performance in both localized heatmap activation and overall detection accuracy. On the one hand, the heatmap activation is more focused around the ureteral orifice and exhibits a circular/ring-shaped distribution, which aligns well with intuitive anatomical expectations. On the other hand, our detection results on the validation set showed the fewest instances of duplicate bounding boxes, missed detections, and false detections, which is expected to provide a better practical user experience in real applications.

**Figure 12:**
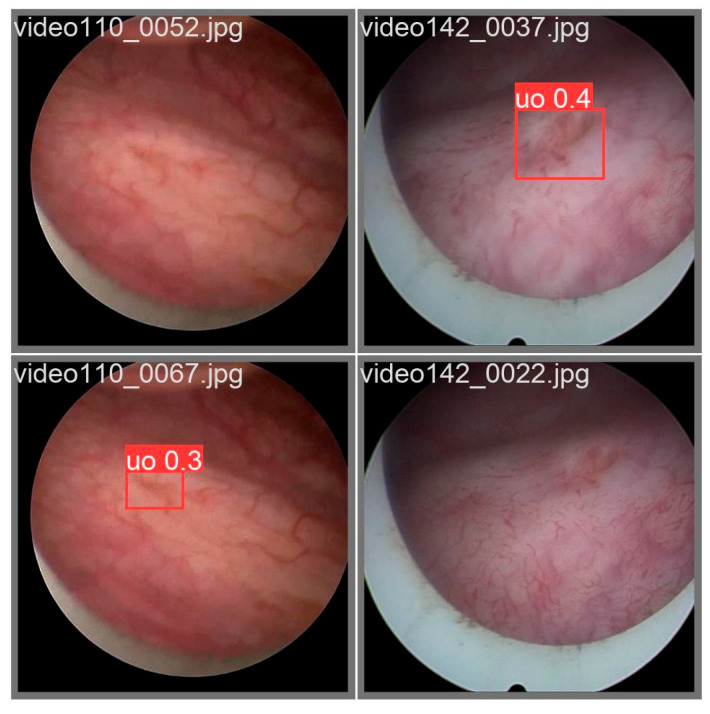
Failure Cases in Object Detection.

Nevertheless, this does not imply that the study is complete; during experimentation, we observed that some validation-set images produced anomalous detection outcomes (see Figure 12). In certain cases, the closed morphology of the ureteral orifice and the blurred image texture rendered detection unstable, leading to intermittent disappearance of bounding boxes in a clinical setting. Future engineering deployments may need to integrate auxiliary methods—such as optical-flow-based tracking algorithms—to compensate for these detection gaps.

At present, the experiments are still exploratory and offer substantial scope for improvement; in particular, ureteral orifices with incomplete morphological structures were not included, limiting the model’s generalizability and representing a key area for future research. Additionally, we lack a standardized framework to quantitatively assess the diagnostic performance of novice, experienced, and expert clinicians in interpreting ureteral orifice images; consequently, the comparative advantage of the model relative to human expertise cannot be accurately demonstrated. In terms of practical deployment, our limited experience in hardware development prevents effective optimization and adaptation of the model to real clinical environments, and this aspect requires further exploration in future studies.

## 6. Conclusions

This study explores the feasibility of ureteral orifice detection in ureteroscopic images based on a large-kernel deep learning network, improving upon a baseline model and achieving favorable performance, thereby providing new references for future research and enabling comparisons with existing studies. However, certain shortcomings persist, and these issues will be the focus of future investigations. The dataset in this research mainly comprises cases without surgical intervention or pathological abnormalities due to ease of data acquisition; however, this approach has limited clinical representativeness, and images depicting lesions or surgery-induced scarring are likely to be emphasized in future studies. In the future, we will endeavor to further enhance the practical applicability of the model, with deployment on prototype platforms compatible with current medical hardware representing a promising optimization pathway and a substantial step toward real-world usability.

In summary, our study demonstrates that using the YOLO model for ureteral orifice detection in ureteroscopic images is an effective approach that could assist physicians in improving medical quality.

## Figures and Tables

**Table 3:** Performance comparison of attention mechanisms.

Attention	*p*-Value	R-Value	mAP@0.5	TP	FP	FN
CASAtt	0.794	0.816	0.862	412	28	79
ECA	0.832	0.846	0.874	430	31	61
ELA	0.850	0.796	0.865	407	27	84
LSKA	0.881	0.798	0.872	433	49	58
CAA	0.861	0.819	0.867	429	26	62
BiFormer	0.841	0.804	0.864	408	13	83
Ours	0.838	0.825	0.890	430	42	61

**Table 4:** Summary of historical datasets.

Number	Case	Train-Figure	Val-Figure
1	85	554	130
2	155	1067	339
3	222	1448	491

**Table 5:** Performance Comparison of Various Object Detection Models.

Attention	*p*-Value	R-Value	mAP@0.5	mAP@0.5-0.95	GFLOPs	Parameters	Variance
YOLOv8	0.852	0.809	0.869	0.376	79.1	25,856,899	0.4956
YOLOv9	0.827	0.799	0.854	0.372	77.5	20,159,043	3.0822
YOLOv10	0.807	0.756	0.830	0.352	64.0	16,485,286	4.0067
RT-DETR	0.793	0.811	0.830	0.338	108.0	32,808,131	0.3489
Ours	0.845	0.813	0.881	0.356	30.5	16,080,166	0.4067

## Data Availability

The data presented in this study are available on request from the corresponding author. The data are not publicly available due to privacy.
